# Present-day tropical precipitation and cloud feedbacks determine future equatorial Pacific trends

**DOI:** 10.1126/sciadv.aea8070

**Published:** 2026-03-06

**Authors:** Samantha Stevenson, Clara Deser, Sloan Coats, Georgina Falster, Browen Konecky, Nicola Maher, Cali Pfleger

**Affiliations:** ^1^ University of California, Santa Barbara, Santa Barbara, CA, USA.; ^2^ National Center for Atmospheric Research, Boulder, CO, USA.; ^3^ University of Hawaii at Manoa, Honolulu, HI, USA.; ^4^ The University of Adelaide, Adelaide, SA, Australia.; ^5^ Washington University of St Louis, St. Louis, MO, USA.; ^6^ Australian National University, Canberra, ACT, Australia.; ^7^ ARC Centre of Excellence for Weather of the 21st Century, Australian National University, Canberra, ACT, Australia.

## Abstract

The equatorial Pacific sea surface temperature (SST) zonal gradient has worldwide impacts and is expected to be highly sensitive to future climate change. However, biases in climate models call the reliability of future SST gradient projections into question. Here, we combine multiple climate model Large Ensembles to show that equatorial precipitation and cloud feedbacks have a controlling influence on the future Pacific SST gradient. An “SST gradient sensitivity” parameter is computed for each model, which shows that models with stronger historical equatorial precipitation have systematically higher sensitivities (more El Nino-like changes). This arises from the stronger negative SST-shortwave radiation feedback, which then creates a wind response that favors El Nino–like warming. Notably, when simulated historical deep convection is sufficiently strong, a “saturation” effect occurs that tends to inhibit this effect. These results imply that models likely underestimate future El Nino–like changes but that the “true” magnitude of changes may be predictable.

## Teaser

Fixing problems with clouds in climate models may make the tropical Pacific more like an El Niño event in the future.

## INTRODUCTION

The pattern of equatorial Pacific sea surface temperature (SST) is critically important for a variety of human and natural systems. The zonal SST contrast (SST gradient) along the equator is particularly impactful because it is closely tied to the location of equatorial deep convection, which alters the propagation of atmospheric circulation anomalies and associated weather patterns around the world (*1*, *2*). The equatorial Pacific SST gradient (hereafter, “SST gradient” or ΔSST) is expected to be strongly modified by ongoing anthropogenic climate change, but the overall magnitude and sign of this effect remains poorly understood (*3*). Therefore, it is critical to quantify the true sensitivity of the Pacific SST gradient to future human influences to improve future projections of climate change impacts.

Coupled climate models generally simulate a weakening of ΔSST in response to greenhouse gas (GHG) increases (*4*). Yet, it is not clear whether this is an accurate representation of the real world as models have known biases in their simulation of equatorial Pacific climate (*5*, *6*), which have been suggested to affect the sensitivity of SST patterns to warming (*7*–*9*). In addition, model simulations of the recent observational period (20th and early 21st centuries) are unable to reproduce the observed tendency for ΔSST strengthening (*10*). Internal climate variability likely contributes to this disagreement, yet recent work indicates that even after accounting for these effects, models are still highly unlikely to simulate the observed recent ΔSST strengthening (*11*).

One difficulty with assessing the accuracy of ΔSST trends in coupled models is our limited understanding of the balance of mechanisms governing their variability and change. Multiple hypotheses have been proposed for the influence of GHG emissions on ΔSST. These include thermodynamic responses in the atmosphere (*12*), the behavior of the oceanic subtropical cells (*13*), changes in mean atmospheric stability (*14*), and differences in the radiative sensitivity of SST across the basin (*15*), among others [see review by Lee *et al.* (*3*) for details]. There is likely to be substantial structural uncertainty in the relative magnitudes of these various mechanisms, but separating structural uncertainty from other forms of uncertainty requires a large number of simulations. Until recently, a suite of simulations of sufficient size has not been available, making it more difficult to draw firm conclusions regarding the influence of model physics on ΔSST changes due to confounding effects from internal climate variability.

Here, we address the question of ΔSST response to 21st century radiative forcing using a suite of “Large Ensemble” (LE) simulations run with a variety of climate models (*16*). An LE is a set of simulations run with a single model, where all simulations are identical with the exception of their initial climate state [e.g., (*17*)]. Over the past 10 years, LEs have been run with many different models and have become widely used in the climate community to separate the forced response from internal variability (*18*, *19*). These LEs now make it possible to directly and robustly examine the differences in models’ forced responses to climate change (determined from the ensemble mean of each LE) and assess their causes. We note that all LEs analyzed here are members of the CMIP6 model generation (tables S1 and S2), which show a higher degree of consistency in the sign of ΔSST change relative to Coupled Model Intercomparison Project phase 5 (CMIP5) (*20*, *21*). This choice was made to prevent complications from arising due to the known differences in climate sensitivity and the treatment of cloud microphysical processes between CMIP5 and CMIP6 models (*22*, *23*).

## RESULTS

### SST gradient sensitivity

**Fig. 1.**
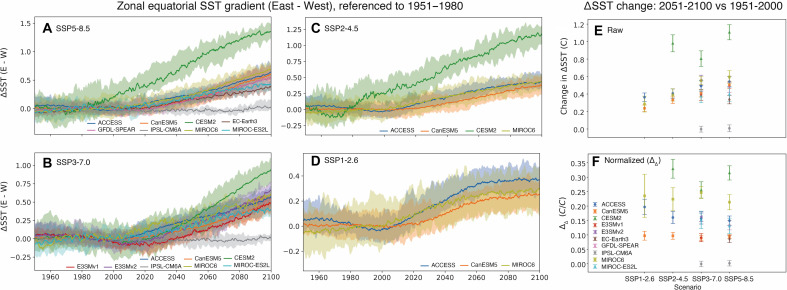
Changes in the SST gradient (ΔSST) in the Pacific. (**A** to **D**) 30-year running mean time series of the SST gradient for four different future scenarios: (A) SSP5-8.5, (B) SSP3-7.0, (C) SSP2-4.5, and (D) SSP1-2.6. Solid lines indicate ensemble median, and colored shading indicates the ensemble minimum/maximum range. All gradient values are calculated relative to the ensemble average over the 1951–1980 reference period. (**E**) Epoch-averaged change in the SST gradient, differenced between 2050–2100 and 1950–2000. (**F**) Same as (E), but normalized to each model’s area-weighted global mean temperature change between the two averaging periods (Δ_Δ_ values, as described in the main text).

The overall response of ΔSST to various future emissions scenarios is summarized in Fig. 1, where the gradient is defined as the difference between temperatures in the eastern and western equatorial Pacific (Materials and Methods). All LEs simulate a weakening of ΔSST over the 21st century, the magnitude of which tends to be larger for higher-emissions scenarios (e.g., compare values in Fig. 1E across scenarios for a given model). However, for a given emissions scenario, the ΔSST change differs between LEs, suggesting a role for intermodel physical differences. For instance, the Community Earth System Model 2 (CESM2) has a much stronger response than other models to the SSP5-8.5 (Fig. 1A), SSP3-7.0 (Fig. 1B), and SSP2-4.5 (Fig. 1C) scenarios. At the other end of the spectrum, the Institut Pierre-Simon Laplace Climate Model version 6 (IPSL-CM6A) exhibits almost no response to either SSP5-8.5 or SSP3-7.0 (the only two scenarios available for this model). There is also some notable nonmonotonicity, primarily in the lower-emission scenarios: All three of the models that ran the SSP1-2.6 scenario level off in their responses between 2060 and 2100 (Fig. 1D) and some indications of similar behavior can be seen under SSP2-4.5 by the end of the century (Fig. 1C). ΔSST changes are summarized as epoch differences in Fig. 1E, where it is immediately obvious that some models’ zonal SST gradient is systematically more responsive to future climate change than others.

The model differences between projections of 21st century gradient changes can be obscured by the differences between emissions scenarios (e.g., Fig. 1E). To allow isolation of the role of intermodel physical differences, it is necessary to normalize the ΔSST time series to their respective global mean temperature increases so that all projections can be compared on an equal footing (Fig. 1F and fig. S1). Some models can then be easily distinguished in terms of their ΔSST sensitivity to global mean temperature increases; for instance, Canadian Earth System Model version 5 (CanESM5) and IPSL-CM6A exhibit little change, while models such as MIROC6 and CESM2 are especially responsive (fig. S1B). The relative magnitude of epoch changes in ΔSST is approximately constant across emissions scenarios for many models (Fig. 1F). One exception is the CESM2, where the response to SSP3-7.0 forcing is proportionally smaller than the response to the other two scenarios. We speculate that this may be related to CESM2’s high climate sensitivity, which has been demonstrated to lead to strong and compensatory responses to GHG and aerosol emissions (*24*). Given that SSP3-7.0 has relatively high aerosol forcing (*25*), this might then create overly strong damping in CESM2. Nevertheless, it appears that, generally speaking, the balance of mechanisms governing ΔSST change at the end of the 21st century does differ systematically between climate models but remains relatively consistent across emissions scenarios for LEs run with a given model.

Using the results of Fig. 1, we define an “SST gradient sensitivity” parameter (hereafter Δ_Δ_) to classify models according to their tropical Pacific responses. The Δ_Δ_ parameter is equal to the value of the change in ΔSST between the 20th century (1951–2000) and 21st century (2051–2100), normalized to the change in global mean temperature over those same periods (Fig. 1F). Where simulations with multiple emissions scenarios are available, these have been used to estimate a scenario-mean ΔSST sensitivity and associated uncertainty (table S3); in subsequent analyses, the ΔSST sensitivities computed for individual model/scenario combinations are used. The results are somewhat sensitive to the start and end points chosen, possibly due to the higher importance of aerosol forcing in the 20th/early 21st centuries (not pictured); here, we use the latter halves of the 21st and 20th centuries to more clearly isolate the effect of GHG emissions (Materials and Methods). To provide some validation of the relative magnitudes of the Δ_Δ_ parameters, we also compute the linear trend in ΔSST over 1950–2100 normalized to the linear trend in global mean temperature and average over all scenarios available for a given model (table S4). This shows qualitatively similar results to the Δ_Δ_ defined using epoch differences.

We also explore the dependence of the Δ_Δ_ parameter on season, since temperature trends may vary as a function of the seasonal cycle (*26*). During JJA, many models exhibit relatively higher Δ_Δ_ values (fig. S2A versus fig. S2B; table S4); these are predominantly the models with lower overall Δ_Δ_ (e.g., ACCESS, CanESM5, and EC-Earth). This seems to relate to the importance of boreal summer in generating changes in western Pacific SST (see the “Future changes associated with SST gradient sensitivity” section).

### Future changes associated with SST gradient sensitivity

**Fig. 2.**
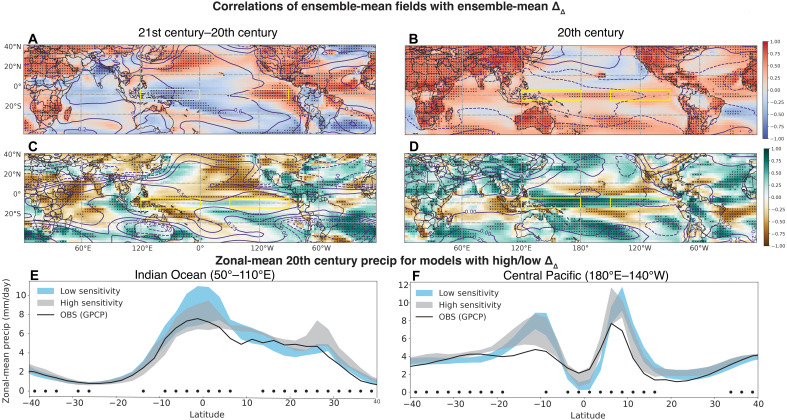
Large-scale circulation patterns associated with Δ_Δ_ spread across models. (**A**) Correlation of ensemble mean gridpoint 21st century–20th century surface temperature (colors) and SLP (contours) differences with the ensemble mean Δ_Δ_, across the collection of all ensemble means for each model/scenario combination. (**B**) Correlation of ensemble mean gridpoint 20th century surface temperature (colors)/SLP (contours) with Δ_Δ_. (**C**) Same as (A), but correlations are performed between gridpoint precipitation (colors) or 500-hPa geopotential height (contours) and Δ_Δ_. (**D**) Same as (B), but correlations are performed between gridpoint precipitation (colors) or 500-hPa geopotential height (contours) and Δ_Δ_. The time-varying global mean is removed from the geopotential height maps before regression to better isolate the spatial pattern. All time differences are calculated between 2050–2099 and 1951–1999, and stippling indicates differences significant at 90% using a Wilcoxon rank-sum test. (**E**) Zonal-mean precipitation averaged over the Indian Ocean (50°E to 110°E), for models with high versus low Δ_Δ_ values. (**F**) Same as (E), but for precipitation averaged over the central Pacific (180°E–220°E). Black dots in (E) and (F) indicate latitudes where the high and low sensitivity models differ significantly at the 90% level using a Wilcoxon rank-sum test. Black solid lines in (E) and (F) show observationally averaged values from the GPCP dataset.

To investigate the mechanisms driving differences in Δ_Δ_, we apply an ensemble mean correlation analysis: This is a previously unidentified approach only possible because of the large number of available ensembles and allows the calculation of spatial patterns most strongly related to the differences between models to be computed. The approach relates the set of ensemble mean Δ_Δ_ values (one value for each model/scenario combination) to the set of ensemble mean patterns of air temperature, precipitation, and other key variables (one spatial map for each model/scenario combination; Fig. 2). For example, for each model/scenario combination, the ensemble mean Δ_Δ_ is calculated, and then the set of Δ_Δ_ values is correlated at each grid point with the set of model/scenario estimates of that grid point’s historical mean air temperature (Fig. 2B) or future change in mean air temperature (Fig. 2A). In addition to surface air temperature, the ensemble mean Δ_Δ_ was regressed against grid point sea level pressure (SLP), precipitation, and geopotential height. The resulting Pearson’s correlation coefficient (*r*) reveals, for the full set of model/scenario combinations, the overall patterns of temperature and atmospheric circulation associated with intermodel differences in Δ_Δ_.

Figure 2 shows that there is a spatially coherent pattern of future changes associated with the ensemble mean Δ_Δ_. By construction, warming in the eastern equatorial Pacific is present, along with cooling over the western Pacific warm pool (Fig. 2A). However, ΔSST reductions are also associated with other regions: Future warming in the North Pacific, the tropical Atlantic, and the North American continent are associated with El Niño–like changes (Fig. 2A). This is verified by comparison with the differences in future-historical SST between models with “high” and “low” Δ_Δ_ values (Materials and Methods and fig. S3), which also show larger relative warming in the same locations mentioned above.

The future precipitation changes associated with ΔSST reductions (Fig. 2C) include an overall reduction in precipitation north of the equator throughout the central/eastern Pacific and a dipolar precipitation signal in the Indian Ocean and tropical Atlantic. In the Northern Hemisphere mid-latitudes, the 500-hPa geopotential height changes include low heights over the Atlantic and much of Eurasia (Fig. 2C). In contrast, the Southern Hemisphere mid-latitude circulation exhibits more zonal structure, with lows over Australia and South America coincident with local surface cooling (Fig. 2C).

**Fig. 3.**
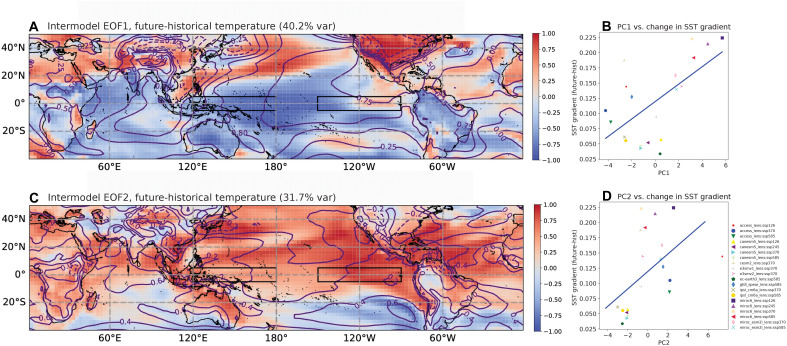
EOF analysis of intermodel spread. (**A** and **C**) Spatial patterns of correlations of temperature (colors) and SLP (contours) with the first two principal components of ensemble mean temperature difference, calculated between 2050–2099 and 1950–1999. Here the ensemble mean temporal differences in each model/scenario combination are treated as individual data points [see also legend next to (D)], and the EOFs are computed along the [model + scenario] dimension, similar to the correlation analyses of Fig. 2. (A) Spatial pattern of mode 1, (**B**) PC1 versus the 21st century–20th century difference in the SST gradient (*R*^2^ = 0.38), (C) Spatial pattern of mode 2, and (**D**) PC2 versus 21st century–20th century SST gradient difference (*R*^2^ = 0.28).

The associations in Fig. 2 are suggestive but may not necessarily represent overall coherent intermodel spread patterns. To verify that this is indeed the case, we also calculate the empirical orthogonal function (EOF) modes of ensemble mean epoch differences in surface air temperature for all ensembles (Fig. 3). The dominant mode (Fig. 3A) explains 40% of the variance in intermodel temperature changes, and the spatial structure strongly resembles the pattern of ensemble mean temperature association with the ΔSST change (Fig. 2A). Features include widespread cooling over the central/west Pacific and Indian Ocean, as well as warming over the North Pacific and Northern Hemisphere land masses. Mode 2 also explains a substantial proportion of variance (31%) and contains similar eastern Pacific/tropical Atlantic signatures to those seen in Fig. 2A. Both modes are significantly correlated with the future-historical SST gradient change (Fig. 3, B and D). These patterns bear some resemblance to the individual effects of GHG and aerosol forcing (not pictured), although the impacts of GHGs are expected to dominate by the end of the century [e.g., (*27*)]. The lack of single-forcing LEs extending beyond 2015, however, prevents this from being systematically explored using the present ensemble mean EOF/regression approach.

To provide additional insight into the mechanisms for intermodel spread, we calculate the correlation of future-historical gridpoint epoch differences with the eastern and western Pacific regions individually (fig. S3). This shows that mode 1 of intermodel spread strongly resembles the circulation pattern associated with western Pacific cooling (fig. S4, A/C versus Fig. 3A) and that mode 2 resembles the correlation pattern associated with eastern Pacific warming (fig. S4, B/D versus Fig. 3C). The western Pacific is more highly correlated with the Indian Ocean than the Atlantic and with strong warming in the North Pacific and over land in the Northern Hemisphere (fig. S4A). A suppression of western Pacific convection due to cooling should drive large-scale subsidence and enhanced easterlies over the Indian Ocean (*28*), consistent with the dipolar precipitation signal near 120°W to 60°W in fig. S4C. In contrast, eastern Pacific warming is related to a more zonally uniform tropical warming pattern, and an equatorward migration of the Intertropical Convergence Zone (ITCZ) (fig. S4, B and D). This may indicate that the eastern Pacific is responding to global mean temperature, with enhanced equatorial warming related to reduced Newtonian damping due to evaporative cooling (*29*).

### Historical patterns associated with SST gradient sensitivity

Another powerful feature of the ensemble mean analysis technique is the ability to relate future changes to the simulated historical climate. This technique enables the identification of aspects of present-day climate with potential predictive power in determining future changes associated with the SST gradient. Fig. 2, B and D correlate historical gridpoint mean climate with Δ_Δ_ for all ensembles. Once again, coherent patterns emerge: Models with larger Δ_Δ_ show higher historical precipitation in the tropical Atlantic, and lower historical precipitation occurs in the Indian Ocean (Fig. 2D). Models with higher Δ_Δ_ also exhibit stronger historical precipitation near the climatological locations of the ITCZ and South Pacific Convergence Zone (SPCZ). High 500-hPa heights over the Tibetan Plateau are associated with large Δ_Δ_, with a wavetrain-like signature present over the mid-latitude North Pacific suggestive of anomaly propagation from the Tibetan Plateau region (Fig. 2D). Historical ocean temperatures in most locations are not significantly correlated with Δ_Δ_ (Fig. 2B), but land temperatures have a strong relationship over the majority of the land surface. The magnitude of the correlation is particularly strong over mid-latitude Eurasia and North America (Fig. 2B) but persists nearly worldwide.

Future cooling in the western Pacific is not strongly correlated with historical ocean temperatures (fig. S5A), but strong associations do emerge with precipitation over the western Pacific and Indian Ocean (fig. S5C). This is consistent with previous work showing that stronger historical western Pacific convection tends to favor El Niño–like warming (*30*). Future warming in the eastern Pacific, however, is associated with warmer historical temperatures throughout the Indo-Pacific and Northern Hemisphere land masses, as well as the North Atlantic (fig. S5B). Little association is seem with the tropical Atlantic, in contrast to previous work showing warm tropical Atlantic conditions favoring El Niño–like warming (*31*). Rather, this pattern suggests that models with higher global mean temperatures also exhibit larger future eastern Pacific warming—the mechanisms for this are discussed further below.

The presence of systematic historical mean-state differences between models with high versus low Δ_Δ_ is further confirmed by separating models into populations based on their Δ_Δ_ values (Methods). In the eastern Indian Ocean, historical precipitation is significantly lower in models with higher Δ_Δ_ (Fig. 2E). In contrast, in the central Pacific, models with higher Δ_Δ_ show larger historical precipitation along the equator and a tendency for reduced off-equatorial precipitation indicative of a southward ITCZ shift (Fig. 2F). The spatial patterns of historical mean-state differences between high- and low-Δ_Δ_ models (fig. S6, A and B) also bear a strong resemblance to those of Fig. 2. Higher-Δ_Δ_ models have higher historical air temperatures over land (fig. S6A), particularly over Eurasia and northern Africa—this effect is largest during December-January-February (DJF) (fig. S6C). The precipitation patterns in the tropics also show a tendency for drier historical conditions over the Indian Ocean and Maritime Continent during DJF (fig. S6D), with enhanced rainfall over the western/central Pacific in all seasons.

We note that the higher historical eastern Pacific temperatures in high-Δ_Δ_ models are more pronounced during JJA (fig. S6E), but the historical land surface temperature is slightly larger during DJF (fig. S6C). The precipitation patterns differ slightly as well, with the subtropical Indian Ocean showing drier historical precipitation during DJF (fig. S6D) and wetter historical conditions over the Indian subcontinent during JJA in high-Δ_Δ_ models (fig. S6F). This difference may relate to influences on the Indian summer monsoon and potentially also to connections with the Indian Ocean Dipole, which peaks in boreal autumn (*32*).

### Cloud radiative impacts on intermodel spread

#### All models

**Fig. 4.**
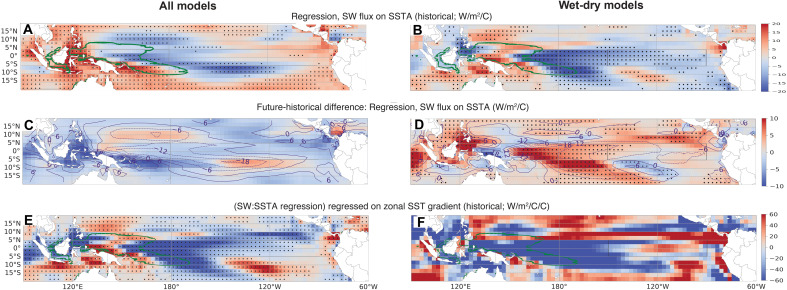
Cloud shortwave feedback relationships. (**A**, **C**, and **E**) Relationships for the set of all ensembles; (**B**, **D**, and **F**) Differences in relationships between the set of all ensembles where central equatorial Pacific historical mean precipitation exceeds 4.5 mm/day, and those where precipitation is below 4.5 mm/day. [(A) and (B)] Multimodel ensemble mean of historical gridpoint regression coefficients for net shortwave flux regressed onto local SST anomaly (regressions computed individually for each ensemble member, averaged over the ensemble, then the multiensemble average computed). Green contour indicates the multimodel ensemble mean climatological location of the 28°C isotherm. [(C) and (D)] Differences between the future-historical SW:SSTA regression coefficient (colors) and the historical values (contours). [(E) and (F)] Regression of the SW:SSTA regression coefficient in (A) onto Δ_Δ_ (stippling indicates locations where regression is significant at the 90% level). Stippling in (A) and (B) indicates locations where two-third of model/ensemble combinations agree on the sign of the regression coefficient.

The previous section demonstrates the clear connection between intermodel spread in historical mean climate and Δ_Δ_, which likely reflects a systematic dependence of atmosphere/ocean feedbacks on mean climate. To further explore this possibility, we calculate the regression of gridpoint net surface shortwave radiation on SST anomalies, where anomalies are calculated relative to the seasonal cycle and the ensemble mean (fig. S7) is removed from each member before analysis (Fig. 4). These regressions are computed over the time dimension for individual ensemble members, then the ensemble mean for each model/scenario combination is averaged (see fig. S8 for the historical regression patterns in individual models), and then the ensemble means are averaged together to form the maps in Fig. 4. Here, these calculations roughly follow the “cloud-shortwave feedback index” approach of (*7*), which was shown to effectively indicate the influence of cloud feedbacks on the SST pattern responses to anthropogenic warming. We note that there is also some influence from longwave feedbacks, which generally have a warming effect in the tropics due to absorption of upwelling longwave radiation by deep convective anvils (*33*, *34*). The magnitude of this feedback at the surface, however, is generally small compared with the shortwave effect (fig. S9 versus fig. S8), and we therefore focus on shortwave feedbacks here for simplicity.

In the tropical oceans, a negative regression coefficient between surface net shortwave flux and SST anomaly (SSTA) (SW:SSTA) indicates that local warming increases cloud cover (and decreases surface shortwave radiative flux), which is generally true in regions dominated by deep convection (see Fig. 4A). Negative coefficients can be seen in cumulus-dominated regions such as the western Pacific warm pool and the tropical Atlantic, and zonal bands of negative coefficients are also present near the climatological locations of the ITCZ and SPCZ (Fig. 4A). The negative coefficients near 5°N to 10°N do not fully persist across the basin in the multimodel mean (Fig. 4A), where coefficients become smaller or positive for longitudes east of roughly 150°W. This may reflect either the presence of stratiform rain within the ITCZ [e.g., (*35*, *36*)] or the diversity of representation of the ITCZ location and strength across models (fig. S8). In contrast, positive SW:SSTA coefficients are present in stratus regimes of the far eastern equatorial Pacific, where SST increases lead to reduced cloud cover and increased surface shortwave radiation.

There is overall low intermodel agreement on the sign of the regression coefficients in the central/eastern equatorial Pacific (Fig. 4A). The multimodel ensemble mean value is generally negative west of 160°W and positive eastward, but less than two-third of models agrees on the sign at any given longitude. This disagreement is an indication of the large spatial diversity of the feedback patterns across models (see also figs. S8 and S9). To assess the importance of this intermodel diversity in generating spread in future projections, we apply another ensemble mean regression: a regression of the SW:SSTA coefficients of Fig. 4A on the Δ_Δ_ for each model/scenario combination. There is a large negative association near 160°E to 200°E along the equator, with a positive association immediately to its west (Fig. 4E). This suggests that models where the historical location of deep convection is shifted farther to the east (or where deep convection is stronger overall) are also those which experience a stronger future weakening of ΔSST.

The sign transition near 140°E in Fig. 4A coincides approximately with the climatological edge of the western Pacific warm pool (green contour). The western Pacific also generally tends to be the location where the equatorial sensitivity of precipitation to SST anomaly is largest, as measured by the regression of gridpoint precipitation anomaly onto local SSTA (fig. S10). Near the warm pool edge, variations in local SSTA are most effective at exciting deep convection, as indicated by both the negative SW:SSTA feedback and the increase in precipitation sensitivity to SST [e.g., (*9*, *37*); see also fig. S8]. In addition, differencing the precipitation:SST anomaly regression coefficients between high and low Δ_Δ_ models shows that high-Δ_Δ_ models have more positive precipitation responses to SSTA in the central Pacific over the 20th century (fig. S11).

**Fig. 5.**
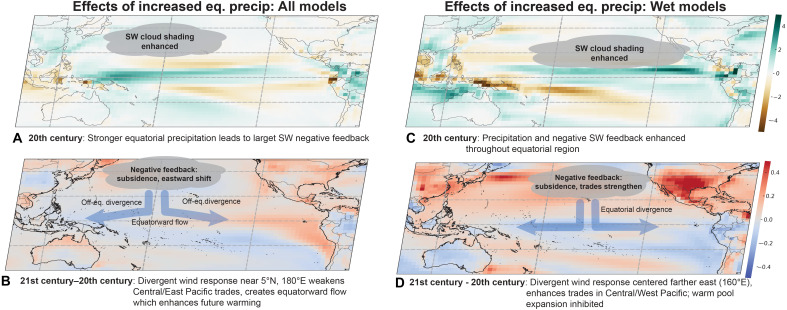
Schematic illustrating the proposed mechanism for El Niño–like ΔSST changes in response to future warming. (**A**) Colors indicate ensemble mean precipitation differences between high and low SST Δ_Δ_ models (mm/day) over the 20th century. (**B**) Colors indicate ensemble mean temperature differences between the future and 20th century, differenced between high and low Δ_Δ_ models (**C**). (C) Same as (A), for differences between sets of wet models: (MIROC6, MIROC-ES2L) – (CESM2, E3SMv2). (**D**) Same as (B), for differences between (MIROC6, MIROC-ES2L) and (CESM2, E3SMv2).

Our proposed mechanism linking the feedbacks above with future SST gradient responses is as follows (Fig. 5). In models with historically stronger precipitation over the central equatorial Pacific, the negative SW:SSTA feedback will be larger in the central/western Pacific, since a smaller increase in SSTA is required to initiate additional convective precipitation in this region. (In the eastern equatorial Pacific, the SST lies generally below the threshold for deep convection, and stratiform clouds with positive SW:SSTA feedback are favored.) The dominance of convection in the central/west Pacific is demonstrated in fig. S12, where it is clear that the threshold for convection [calculated following (*38*)] is exceeded the vast majority of the time throughout the region. Negative shortwave feedbacks associated with a convective regime will tend to suppress local CO_2_-induced warming and induce an anomalous lack of further convection. The resulting anomalous atmospheric descent operates essentially as the inverse of the classical “Gill” (*39*) response to surface heating: Descending air near the dateline will tend to diverge at the surface and induce a westerly wind anomaly. The region where this effect is most important actually lies slightly north of the equator (fig. S13), near the climatological position of the ITCZ. High-Δ_Δ_ models exhibit a weakening of the ITCZ, especially during boreal summer, which favors northwesterly flow onto the equator in the central Pacific and leads to a more El Niño–like wind pattern (fig. S13, E and F).

The above mechanism is conceptually similar to that proposed by (*7*), who proposed that systematic underestimates in the strength of SW:SSTA feedbacks should cause erroneous surface convergence and cause models to simulate overly La Niña–like responses to GHG increases. Here, we show that models with high Δ_Δ_ behave in essentially the opposite fashion: when convection is stronger, surface divergence favors El Niño–like warming. Crucially, the location of the divergent response differs between this study and (*7*), as the largest signal is generally north of the equator in this analysis.

#### Wet models

The relationships shown in Fig. 4 change in models with extremely high climatological mean equatorial precipitation (Materials and Methods). In these models (hereafter the “wet” models; table S6), the negative SW:SSTA relationship is significantly enhanced relative to “dry” models with weaker climatological precipitation (see stippling in Fig. 4B). However, in wet models, the future-historical change in this feedback is significantly more positive than for dry models (Fig. 4D), whereas for models as a whole, the tendency is for the negative SW:SSTA feedback to become more negative in the future (Fig. 4C). This suggests that for the wettest models, the divergent wind response may be expected to be more active than in drier models, albeit while weakening in the future. The pattern of SW:SSTA regressions associated with Δ_Δ_ also differs in wet models (Fig. 4F), with much larger negative signals over the far western Pacific and larger positive signals over the far eastern Pacific.

**Fig. 6.**
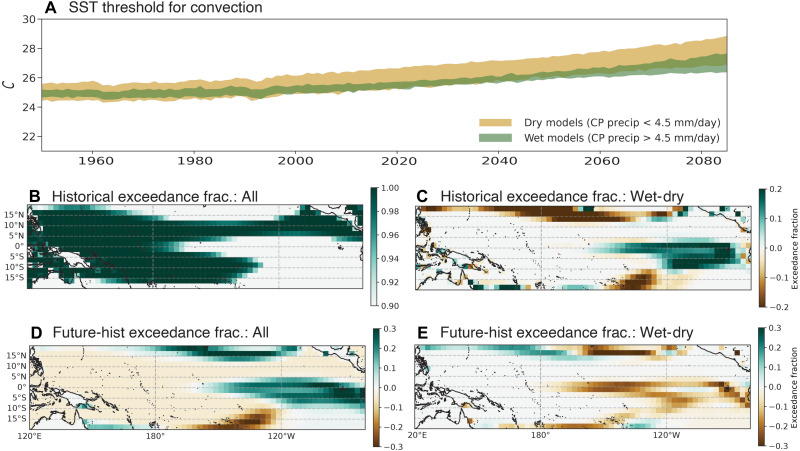
Convective threshold behaviors. (**A**) Time series of the SST threshold for deep convection (tropical precipitation ≥ 2 mm/day), for the dry and wet subsets of model ensembles. (**B**) Multimodel ensemble mean convective threshold exceedance fraction, averaged over the 20th century. (**C**) Difference between convective threshold exceedance fraction, for the wet versus dry models. (**D**) Difference between the multimodel ensemble mean exceedance fractions, between the future and 20th centurys. (**E**) Difference between the future-historical exceedance fraction differences, between the wet and dry model subsets.

We hypothesize that this regime transition from large historical negative feedback to future warming relates to the “saturation” of atmospheric convection at high SST values. Wet models generally begin with warmer SSTs in the eastern Pacific over the 20th century (fig. S14A), which then results in a mean equatorward shift of the ITCZ that favors stronger equatorial precipitation (fig. S14, B and D). These models also seem to be inherently more convectively active along the equator, likely due to differences in their treatment of convective parameterizations: This difference is evidenced by their systematically convective SST thresholds (Fig. 6A and Materials and Methods). This means that a higher proportion of the tropical ocean exceeds the convective threshold in these models (Fig. 6C). However, the exceedance fraction in wet models is less likely to increase in the future (Fig. 6E versus 6D), since convection is already commonplace in the 20th century in these models. This effect can be observed both for wet models as compared with dry models (Fig. 6E) and within the population of wet models (fig. S15).

The mechanism driving this behavior for the wet models is summarized in Fig. 5 (C and D). The initially large central equatorial Pacific cloud shading drives a divergent wind response in terms of future-historical changes, as is the case for the full population of models. However, because the wet models have warmer eastern Pacific SSTs (fig. S14), deep convection can initiate in the eastern portion of the basin. This shifts the location of strong SW:SST feedbacks in these models farther east (see green-outlined panels in fig. S8). In addition, convection along the equator is stronger in these models, meaning that the divergent wind response to warming is centered more closely along the equator (fig. S16, D to F). This response is strong along the equator throughout the calendar year, and the center of action falls near 200°E, east of the climatological warm pool edge. This makes it more difficult for warm pool water to be advected east, suppressing the Bjerknes feedback and inhibiting further El Niño–like warming.

### Implications for constraining future projections

**Fig. 7.**
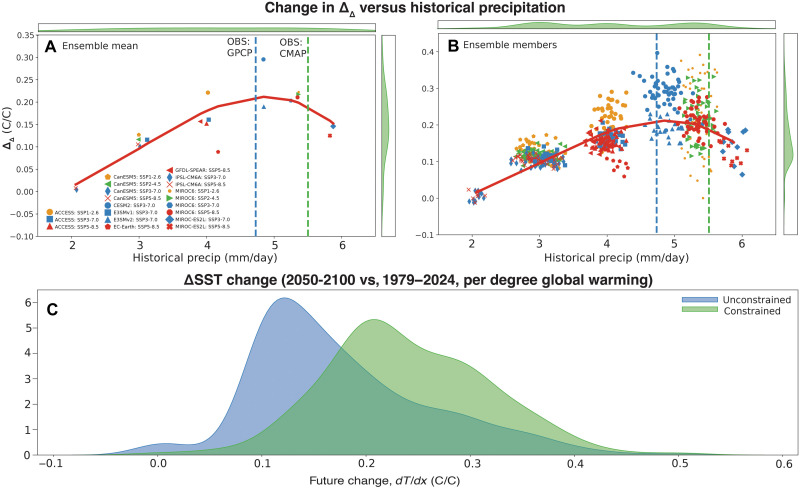
A proposed emergent constraint on ΔSST. The Δ_Δ_ value is plotted versus the historical average of central Pacific precipitation (5°S to 5°N, 150°E to 150°W). Here, the 20th century is defined as 1979–2024 to maximize overlap with observations, and the future period is defined as 2050–2100. Vertical dashed lines indicate the historical precipitation changes derived from observations: GPCP (blue) and CMAP (green). Solid lines of best fit are plotted in red, calculated using a third-degree polynomial spline. Plots along *x* and y axes of (**A**) and (**B**) indicate kernel density estimate (KDE) fits to the distribution of precipitation (*x* axis) and temperature gradient change (*y* axis). (**C**) Emergent constraint applied to ensemble-member estimates, along with the distribution of unconstrained values. To derive the constrained distribution, all ensemble members in (B) where historical (1979–2024) precipitation falls between the GPCP and CMAP estimates are selected, and then the KDE for the corresponding Δ_Δ_ over (2050–2100) – (1979–2024) values is constructed.

The concept of “emergent constraints” based on historical observations that can be used to predict the reliability of future projections has recently been applied in various contexts, including equilibrium climate sensitivity, global mean temperature, and runoff (*40*–*42*). The strong relationship between historical convective precipitation and Δ_Δ_ suggests that this framing may also be useful here. Motivated by Fig. 2, we relate Δ_Δ_ to the historical mean precipitation in the central Pacific (5°S to 5°N, 150°E to 150°W; Fig. 7A). This region is chosen since it coincides with the large correlation between Δ_Δ_ and historical precipitation in Fig. 2D. The fits in Fig. 7 are performed using a spline polynomial, since the sign of the relationship reverses for historical precipitation values greater than roughly 4.5 mm/day. This results in improved estimates of explained variance relative to a simple linear regression (not pictured). The fitted relationship is highly statistically significant, and the observations lie within the spread of the models. However, both the Global Precipitation Climatology Project (GPCP) and Climate Prediction Center Merged Analysis of Precipitation (CMAP) estimates are on the high end of the simulated values, suggesting that many models tend to underestimate historical precipitation in this region (see also fig. S17). This is also consistent with the known cold-tongue bias in coupled models, which suppresses deep convection in the central equatorial Pacific (*5*); however, we again note that convective activity does persist throughout the western and central Pacific, even in models with stronger cold biases (fig. S12).

The fact that the observational estimates of precipitation coincide with the reversal of the precipitation:Δ_Δ_ relationship in Fig. 7 is notable. This study cannot determine the precise reasons for this correspondence, which is left for future work. However, we note that a threshold of 5 mm/day for equatorial Pacific precipitation has been previously shown to be appropriate for identifying large-scale convective reorganization (*9*, *43*, *44*). This provides a physical explanation for the existence of the inflection point in Fig. 7 and suggests that such a relationship may truly exist in nature.

Because observations by necessity contain only a single realization, a more fair model/observational comparison uses individual ensemble member estimates. This is shown in Fig. 7B: The scatter is of course larger, but the historical-future relationship remains statistically significant and the explained variance is nearly identical (not pictured). The distinct downward tendency in the relationship at higher precipitation values is apparent, although we note that this is dominated by simulations using Model for Interdisciplinary Research on Climate version 6 (MIROC6) and MIROC-ES2L (Fig. 7B). Nonetheless, the statistical significance of this relationship implies that it may be useful as a true emergent constraint on model projections. We have applied this constraint in Fig. 7C by selecting all ensemble members where the mean precipitation over 1979–2024 falls between the estimates from GPCP and CMAP; the distribution of Δ_Δ_ values in these members are then plotted. The peak of the distribution lies roughly at a gradient change value of 0.2°C/°C global warming, which should be expected to be the “true” change if this relationship holds in nature.

## DISCUSSION

The effect of future climate change on the east-west gradient in equatorial Pacific SST (ΔSST) is of fundamental importance for improving projections of weather extremes and ecosystem impacts. However, the combination of physical differences among models and internally generated climate variability has made it difficult to determine why models project these different futures. Here, we have leveraged the power of combining many LEs to gain insight into the mechanisms underlying intermodel spread in ΔSST. This is an analysis that was not possible to perform even a few years ago—it is only through the combination of many LEs with differing model physics that one can robustly examine changes in the forced response. The results demonstrate the presence of coherent patterns in atmospheric conditions associated with intermodel SST gradient sensitivity (Δ_Δ_; the change in the east-west equatorial SST temperature difference, normalized to global mean temperature change), which also strongly resemble the dominant modes of intermodel temperature spread diagnosed via EOF analysis. This implies that Δ_Δ_ is a robust feature of intermodel circulation differences.

A crucial aspect of our results is the ability to relate Δ_Δ_ and the representation of historical climate. El Niño–like future SST gradient reductions are preferentially associated with larger historical precipitation in the central equatorial Pacific, and smaller precipitation over the Indian Ocean. Enhanced land surface warming is also associated with high Δ_Δ_—warming appears across the majority of the Northern Hemisphere land area, particularly over Eurasia. This suggests that land temperature–driven hemispheric energy contrasts are altering the mean location of the ITCZ (fig. S6), which has further influences on the structure of tropical Pacific radiative feedbacks.

The historical representation of equatorial deep convection seems to drive the emergent future behavior of the SST gradient through modifying the strength of equatorial shortwave:SST feedbacks. Central Pacific feedbacks generally dominate: In models where the location of deep convection is shifted farther east and/or the magnitude of convection is stronger near the dateline, the negative feedback between SSTA and net shortwave flux is larger. This will tend to suppress GHG-induced warming, leading to a relative cool anomaly. The associated anomaly in atmospheric circulation is toward vertical descent and surface wind divergence, a Gill-type response to a relative lack of tropical convection (*39*). When the set of all models is considered, the divergent wind response to warming appears, on average, just north of the equator, reminiscent of an ITCZ weakening. Although the mechanisms for intermodel spread may be distinct from those driving mean climate in a given model, the behavior and position of this divergent response seems to be crucial here.

The above mechanism is modified in models for which the central equatorial Pacific precipitation ≥ 4.5 mm/day (the wet models). In these models, stronger historical precipitation leads to a less El Niño–like pattern. This relates to the initial very strong negative shortwave feedback, which extends across much of the Pacific and is stronger along the equator than in other models. This results in the cloud shading-driven divergent wind response to be centered along the equator and relatively far east, such that wind anomalies are easterly near the warm pool edge. The net effect is a reduced efficiency of the El Niño–like wind response, as compared with the wider model suite where off-equatorial divergence drives northwesterly flow across the basin and enhances El Niño–like changes.

We have used the historical-future climate relationships emerging from the LEs to identify possible observable emergent constraints on Δ_Δ_. Given the strong relationship with the SW:SSTA feedback strength, the magnitude of this regression is a candidate predictor; however, there are large observational uncertainties in flux products that make this less well suited as an emergent constraint (*7*). The historical magnitude of central equatorial Pacific precipitation is more reliably observed and shows a highly robust relationship with Δ_Δ_, with wetter models producing more El Niño–like future changes. Crucially, this relationship is present both among ensemble mean and ensemble member values and allows for direct comparison with values estimated from historical observations. However, the relationship is nonlinear—for the wettest models, the sign of the relationship reverses due to the changes in feedback structure mentioned above.

The inflection point in the emergent constraint relationship occurs close to the observed value of historical precipitation, suggesting that whether models under- or overestimate precipitation, they tend to be insufficiently El Niño–like in their future SST gradient projections. The majority of models underestimate central equatorial Pacific precipitation, and therefore our results would predict that they also underestimate El Niño–like warming. For the wet models that overestimate precipitation, given the negative sign of the relationship for that parameter space, the emergent constraint relation would also indicate that those models should project a more El Niño–like change. In other words, future projections “should” be even more El Niño–like.

Our finding that models are underpredicting El Niño–like changes contrasts with recent studies highlighting models’ inability to reproduce recent La Niña–like trends in the real world. For instance, Wills *et al.* (*11*) analyze many of the same LEs used here and find that even when accounting for internal variability, all models are extremely unlikely to generate 40-year trends in agreement with observations. In addition, recent high-resolution simulations with the CESM1 show an increased level of agreement with observational trends (*45*), suggesting that model resolution may also be important. We hypothesize that although biases in cloud feedbacks seem to favor an underestimation of El Niño–like warming, there may be other processes missing in models, which could generate additional errors that would lead to a prediction of a more La Niña–like mean state. A full accounting is beyond the scope of the present study, but one example might be small-scale ocean variability (e.g., mesoscale eddies/tropical instability waves). The behavior of the mean thermocline may also play a role but could not be analyzed here owing to limitations in data availability.

The fact that 140°E to 160°E is the longitude range where feedbacks relate most strongly to Δ_Δ_ contrasts somewhat with previous literature. For instance, Xie *et al.* (*29*) found that ocean dynamical heating is largest in the western Pacific, while dynamical cooling occurs in the east. A more eastern Pacific–centered response was also found by Stuecker *et al.* (*13*) and Heede *et al.* (*46*) to be induced by the subtropical cells in advecting warmer subtropical waters onto the equator. Here, we show that the central Pacific also has a role to play, being the region with the largest uncertainties in the magnitudes of cloud:SST feedbacks. This longitude range has also been identified as important for cloud radiative feedbacks in previous studies (*7*), but the off-equatorial influences are here shown to play an important role as well. Our results do not preclude the action of previously identified mechanisms but highlights the complexity of the dynamics at play throughout the equatorial Pacific.

These results have important implications for the accuracy of future SST gradient projections. Previous work has suggested that models may simulate overly La Niña–like future SST gradient changes due to underestimation of the SW:SSTA feedbacks (*7*). Our results are consistent with this picture: The “high-Δ_Δ_” models with more El Niño–like gradient changes are also the models that agree more closely with precipitation observations. However, we caution that the possibility of compensating errors still remains and that models may be missing additional processes that might create more La Niña–like gradient projections if they were corrected. Nonetheless, this analysis provides a framework for constructing dynamically based emergent constraints on future tropical Pacific changes and, eventually, possibly a path toward improving the accuracy of future projections.

## MATERIALS AND METHODS

The equatorial SST gradient ΔSST) is defined as the difference between SST in the eastern (5°S to 5°N, 210°E to 270°E) and western (5°S to 5°N, 120°E to 180°E) Pacific. This definition follows (*11*, *19*). These boxes are shown in all map figures in the main text and supplement.

The periods of interest used to define the SST gradient sensitivity parameter Δ_Δ_ are chosen as the “20th century” (1951–2000) and “21st century” (2051–2100). The one exception is in the calculation of emergent constraint diagnostics, where historical values are computed over the 1979–2024 satellite era to maximize the accuracy of comparisons with observations.

The populations of models having high versus low Δ_Δ_ are defined on the basis the distribution of the Δ_Δ_ parameter across model/scenario combinations. Ensembles (model/scenario combinations) where the Δ_Δ_ change is larger than the 60th percentile are considered high sensitivity; ensembles where the Δ_Δ_ change is smaller than the 40th percentile are considered low sensitivity. Results are insensitive to the exact choice of threshold percentiles (not pictured).

Models with the highest (≥4.5 mm/day) climatological historical mean precipitation over the central equatorial Pacific (150°E to 150°W, 5°S to 5°N) are referred to as wet models. Within that model population, the “wettest” models are identified as the upper 50% (MIROC6 and MIROC-ES2L) and differenced relative to the remaining models (CESM2 and E3SMv2) in Fig. 4 (C and D).

The method of Johnson and Xie (*38*) is used to identify the minimum tropical SST associated with convective initiation. For the global tropical (20°S to 20°N) ocean, SST is binned into 0.1°C intervals, and the precipitation coinciding with each SST bin is calculated. The convective threshold is the minimum SST for which precipitation has exceeded 2 mm/day for four consecutive bin values, which is computed as a function of time since the convective threshold is known to increase under global warming (*38*).
