# Supplementary material for: Present-day tropical precipitation and cloud feedbacks determine future equatorial Pacific trends
Source: Sci Adv. 2026 Mar 6;12(10):eaea8070. doi: 10.1126/sciadv.aea8070 (PMC12965325; doi:10.1126/sciadv.aea8070)
Supplement: Supplementary file 1 — Figs. S1 to S17 Tables S1 to S6 [file sciadv.aea8070_sm.pdf]

Science Advances
AAAS

Supplementary Materials for

Present-day tropical precipitation and cloud feedbacks determine future equatorial Pacific trends

Samantha Stevenson *et al.*

Corresponding author: Samantha Stevenson, sstevenson@ucsb.edu

Sci. Adv. **12**, eaea8070 (2026)
DOI: 10.1126/sciadv.aea8070

This PDF file includes:

Figs. S1 to S17
Tables S1 to S6

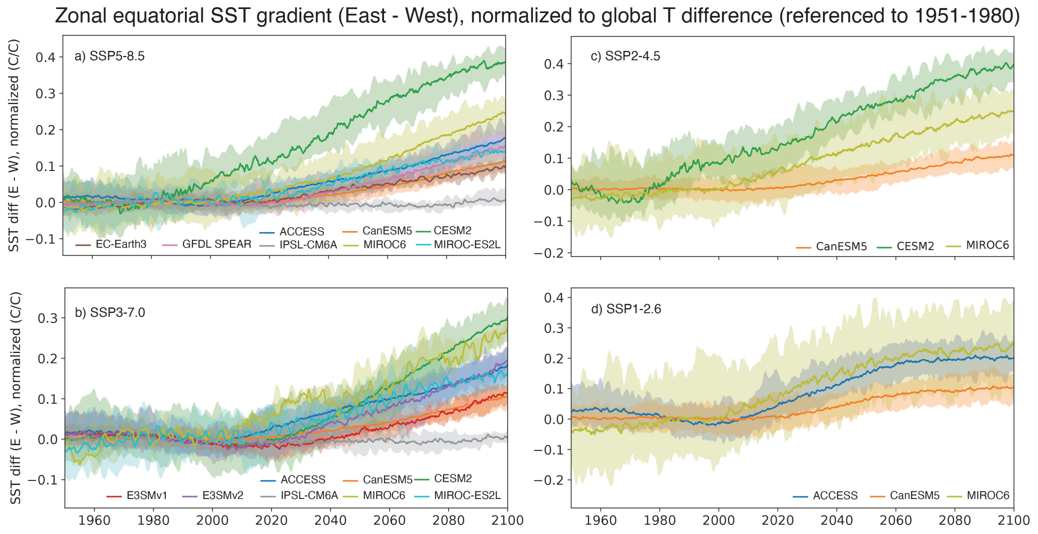

Figure S1: **Running 30-year mean ΔSST time series for SMILEs run under different emissions scenarios: a) SSP5-8.5; b) SSP3-7.0; c) SSP2-4.5; d) SSP1-2.6.** All ΔSST time series have been normalized to the ensemble-mean global temperature change between 2050-2099 and 1951-1999 prior to plotting. Solid lines indicate the ensemble median, and shaded envelopes the ensemble min/max. The normalized ΔSST difference between the 1951-1990 reference period has been subtracted from all time series to ensure overlap between ensembles.

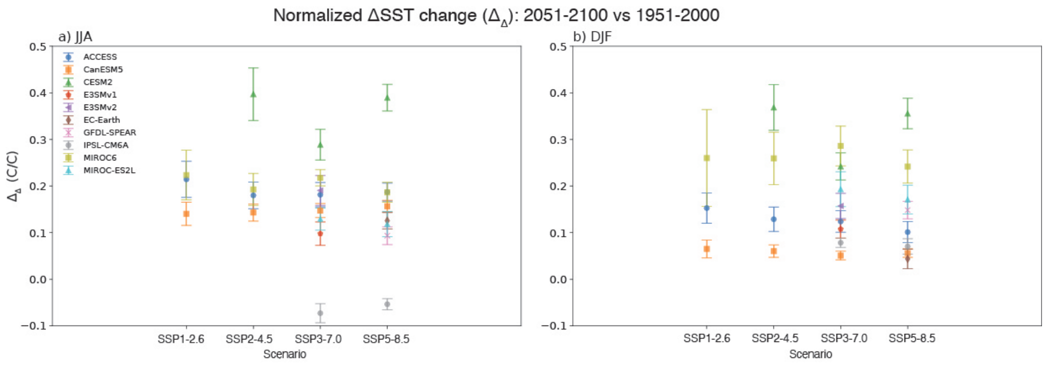

Figure S2: **Seasonally varying Δ_Δ parameter.** Same as Figure 1e,f in the main text, but computed using a) JJA and b) DJF seasonal averages for both the equatorial Pacific SST gradient and the global-mean temperature used in the normalization process.

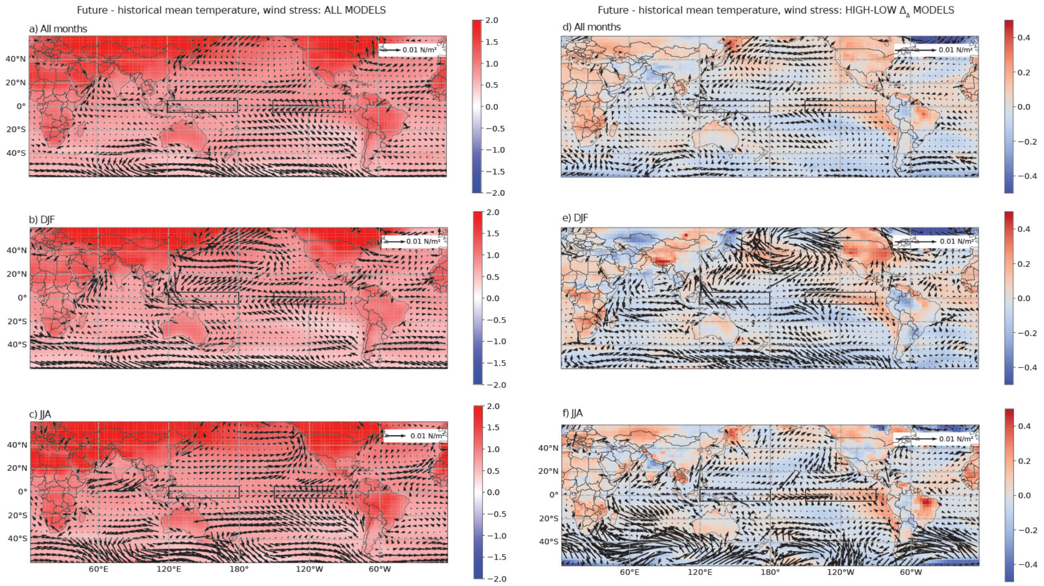

Figure S3: **Mean-state changes for high and low Δ_Δ models.** a)-c): Multi-ensemble average future-historical change in surface temperature (colors) and wind stress (vectors), averaged over all models for a) all calendar months, b) DJF, and c) JJA. d)-f): Same as a)-c), but future-historical changes are differenced between the populations of high and low Δ_Δ models ('High-Sens' and 'Low-Sens' models in Table S6).

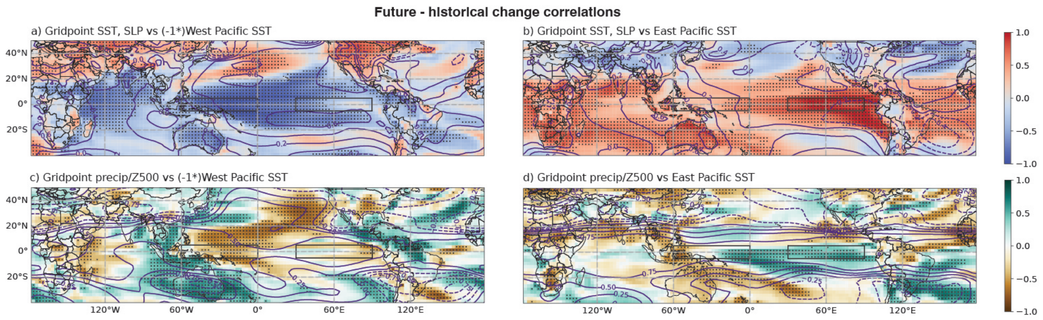

Figure S4: **Future-historical change patterns associated with temperature changes in the east and west Pacific regions.** a), b) Gridpoint future-historical changes in SST (colors) and SLP (contours) correlated with regionally averaged SST changes in the west and east Pacific regions, respectively. c) d) Same as a), b) but for gridpoint precipitation (colors) and 500 hPa geopotential height (contours). Signs of correlations in a), c) have been reversed relative to b), d) in order to show the patterns associated with western Pacific SST cooling.

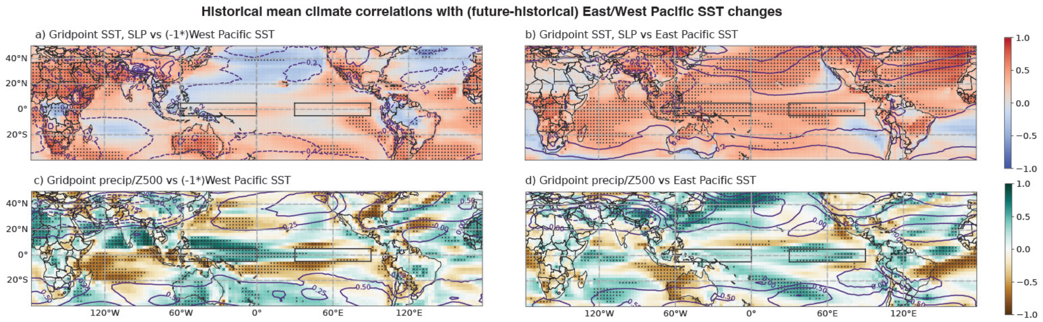

Figure S5: Same as Figure S4, except gridpoint fields are historical averages rather than future-historical differences.

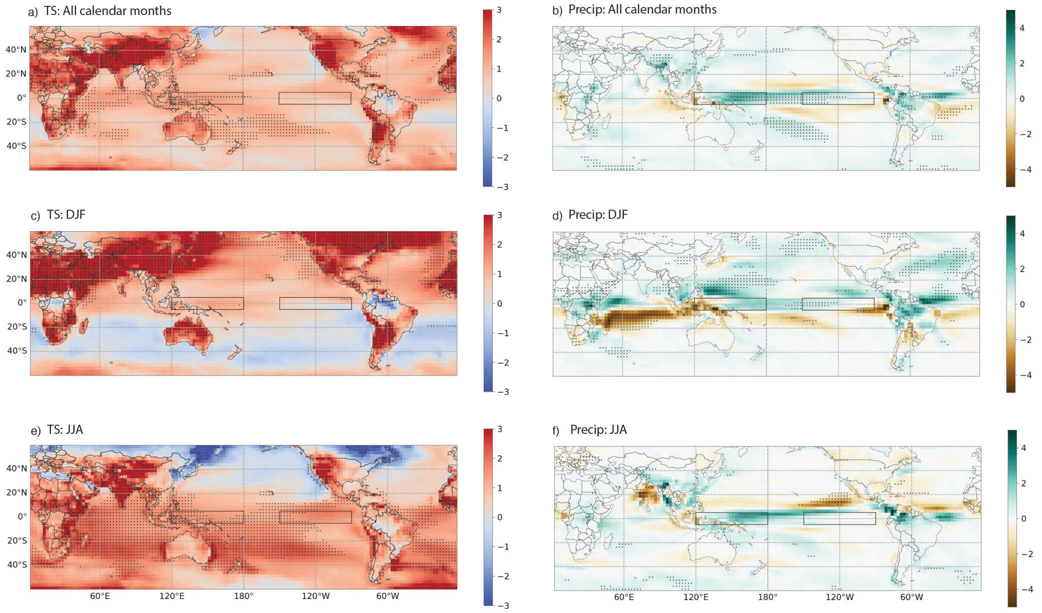

Figure S6: **Differences in 20th century mean climate between models with high and low Δ_Δ.** a) Surface air temperature (C), for all calendar months. b) Precipitation (mm/day), for all calendar months. c), d) Same as a), b) for DJF. e), f) same as a), b) for JJA. "High" sensitivity models are those with ensemble-mean Δ_Δ above the 60th percentile of the set of all ensembles; "low" sensitivity models have Δ_Δ below the 40th percentile. All Δ_Δ values are computed over the same averaging season that was used for mean climate. Black stippling indicates locations where the differences between high and low sensitivity models are significant at the 95% level using a Wilcoxon rank-sum test. 20th century values are calculated over the 1950-1999 period, as in the main text.

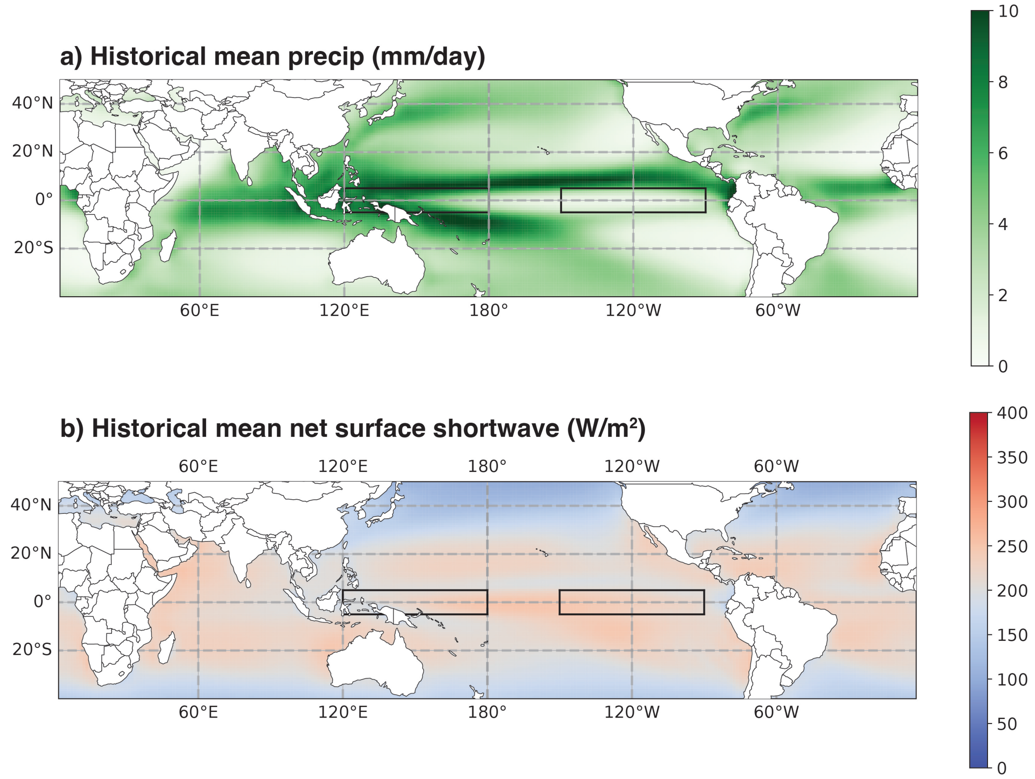

Figure S7: **Mean state averages**. 20th century mean values of a) precipitation and b) net surface shortwave radiation, averaged over all model ensembles.

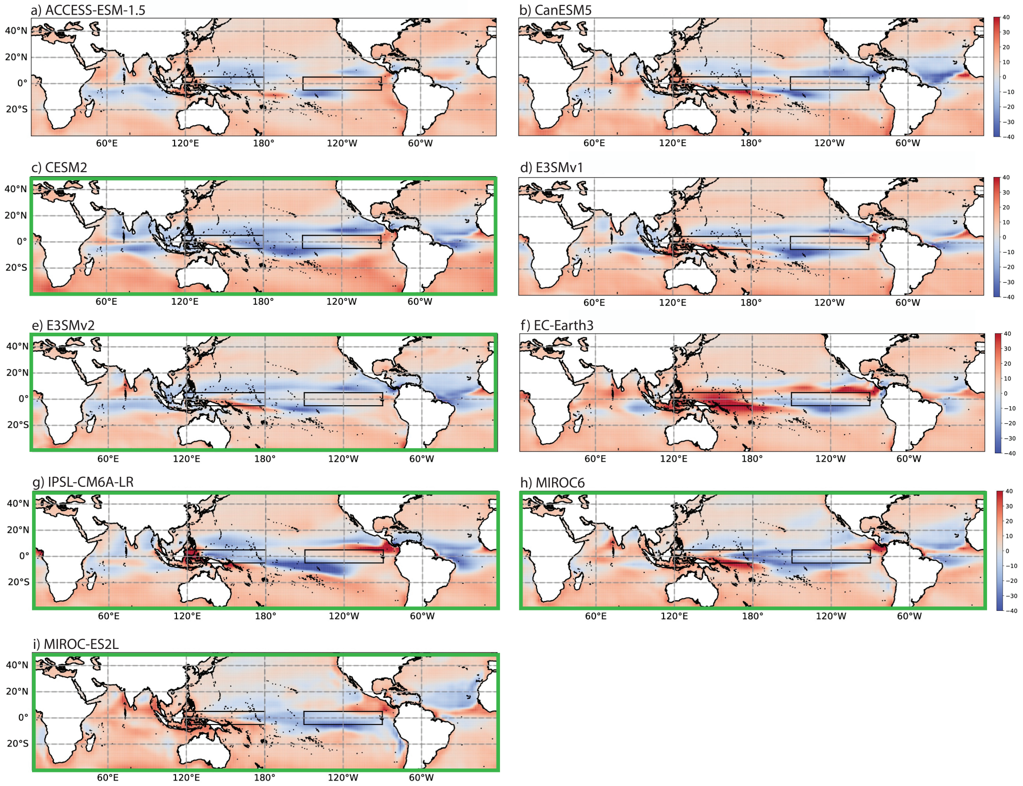

Figure S8: **Regressions of net shortwave flux onto SSTA anomaly for individual ensembles used in the present analysis.** Green bold panel outlines indicate models where 20th century climatological mean equatorial Pacific precipitation exceeds 4.5 mm/day (see Figure 6 in main text).

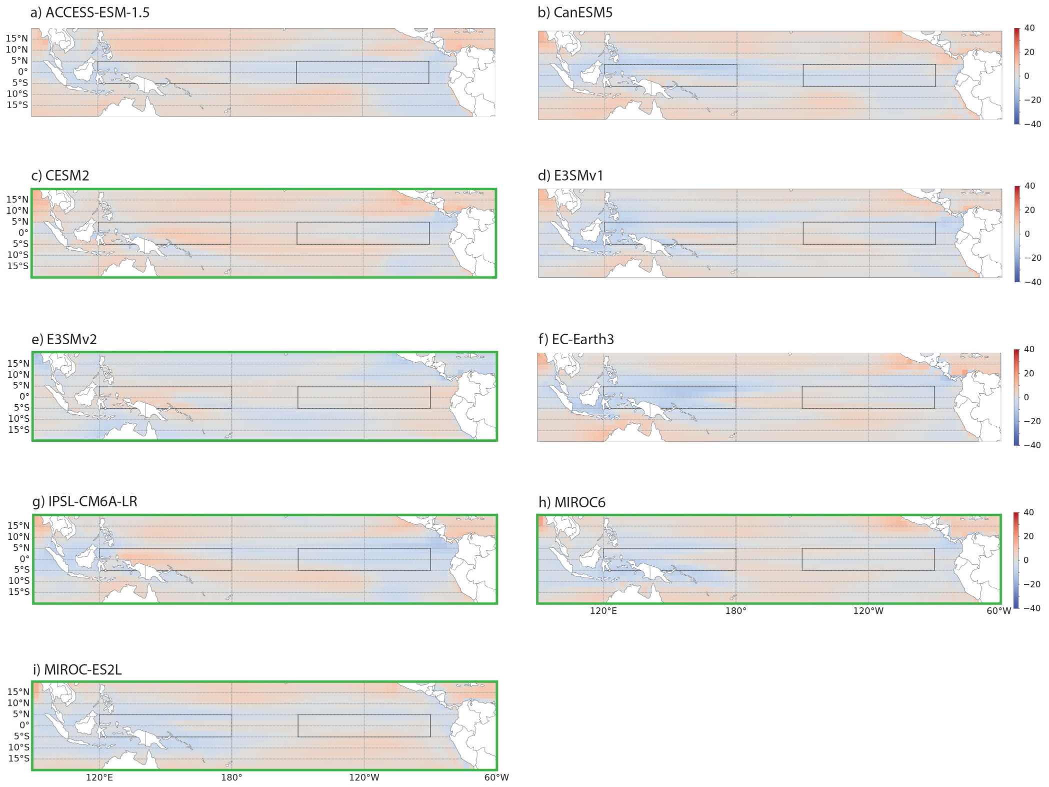

Figure S9: Same as Figure , for net longwave flux regressions onto SSTA.

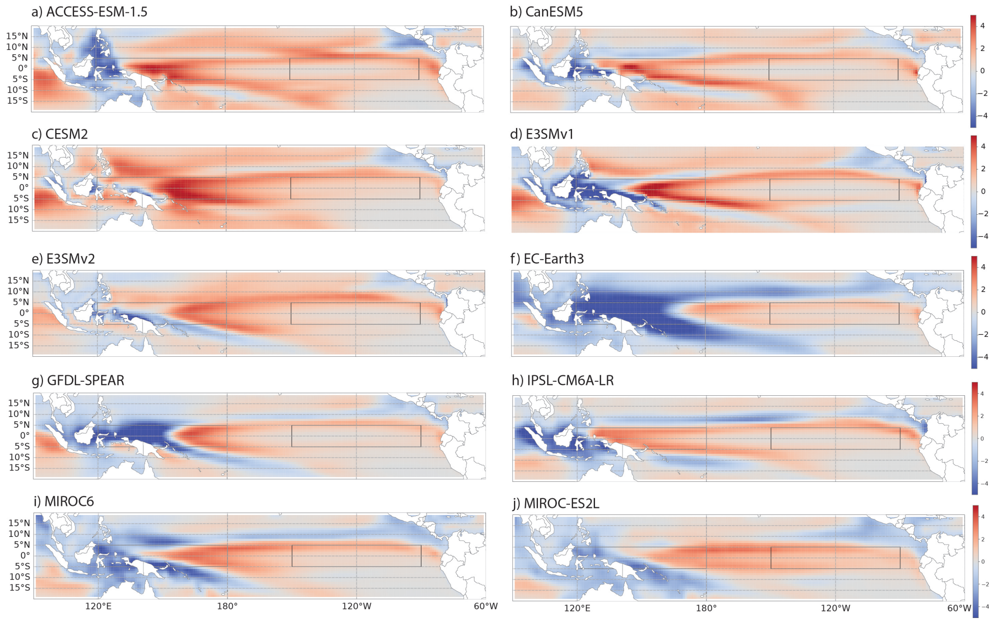

Figure S10: **Sensitivity of precipitation to SSTA**. This is diagnosed by gridpoint regression of precipitation anomaly on SSTA during the 20th century for each individual model included in the present analysis.

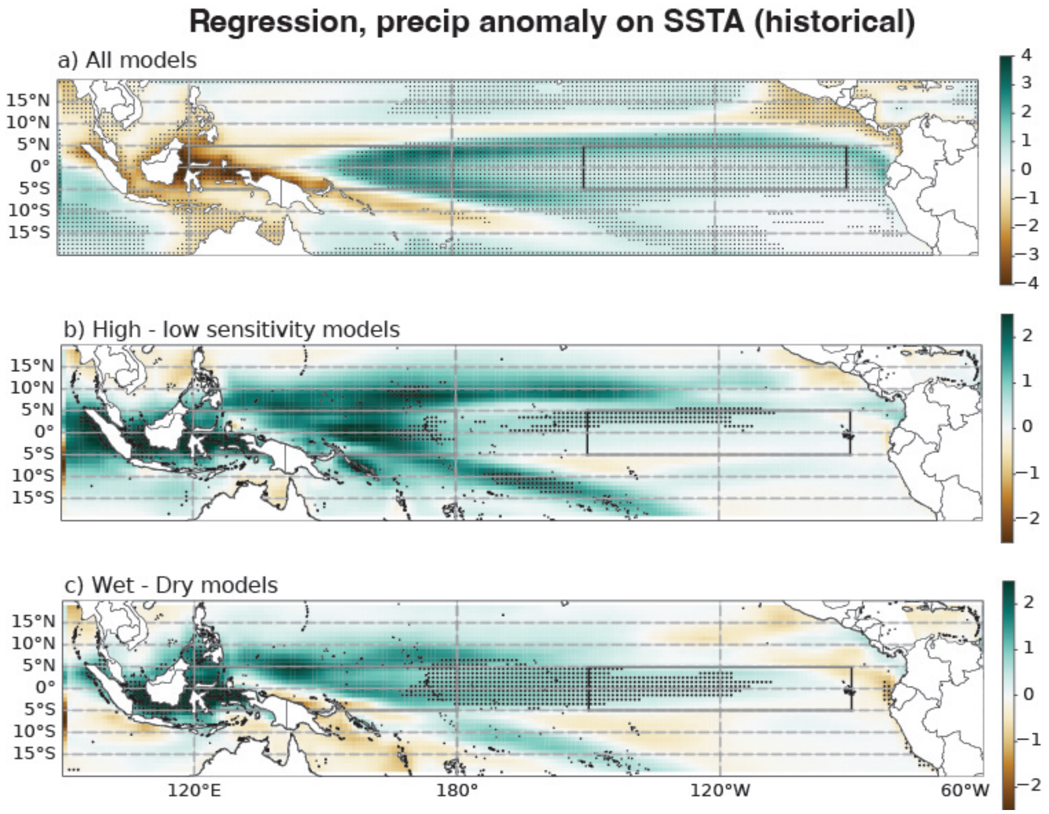

Figure S11: **Sensitivity of precipitation to SSTA**. This is diagnosed by gridpoint regression of precipitation anomaly on SSTA during the 20th century. a) Multi-ensemble mean for all models. Stippling indicates that 2/3 of model/scenario combinations agree on the sign of the regression. b) Difference between high and low-sensitivity models. c) Difference between wet and dry models. Stippling in b) and c) indicates that model populations (high/low sensitivity in b, wet/dry in c) differ significantly at the 90% level according to a Wilcoxon rank-sum test.

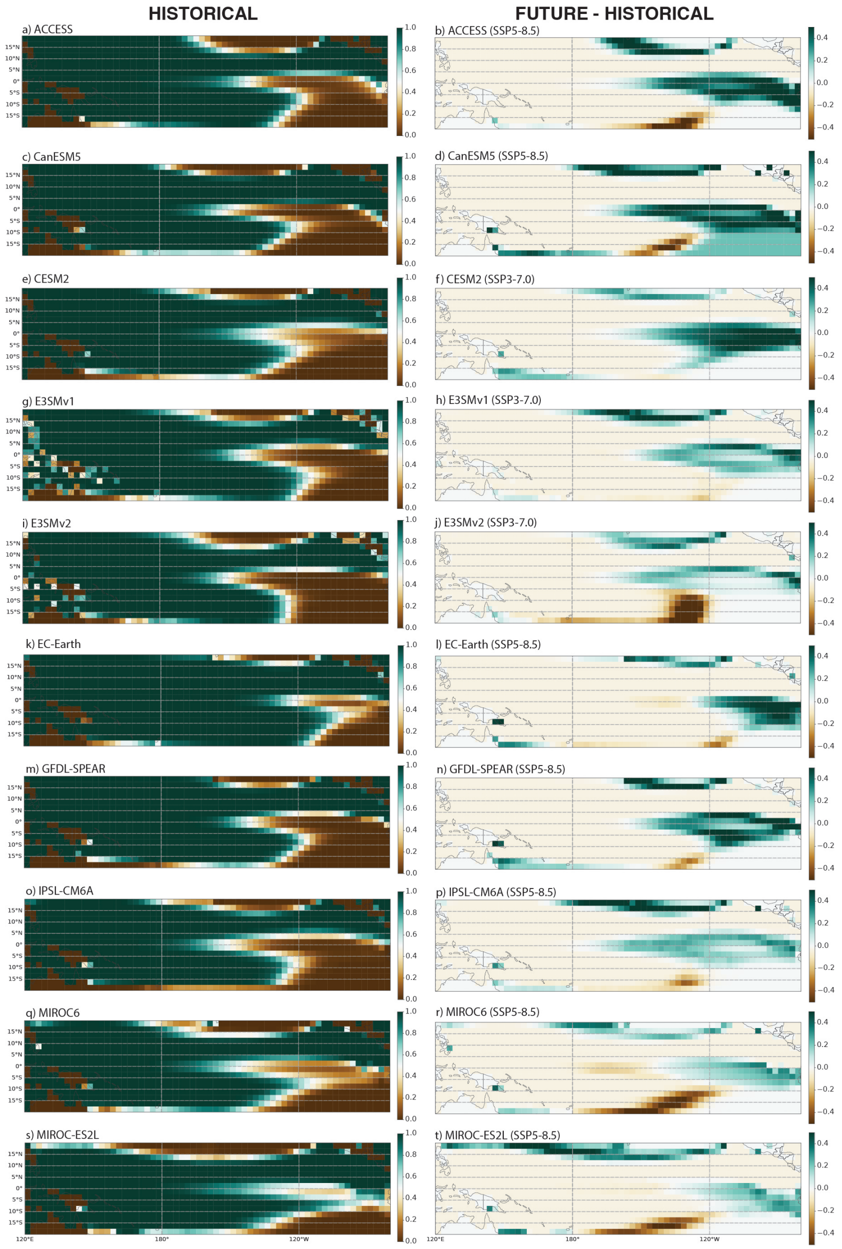

Figure S12: **Fraction of convective exceedances**. For each model in the present analysis, the method of Johnson & Xie (2010) is employed to compute the fraction of time SSTs at each grid point exceed the convective threshold over the historical period (left column), and the exceedance fraction between the future and historical periods for selected SSP scenarios(right column).

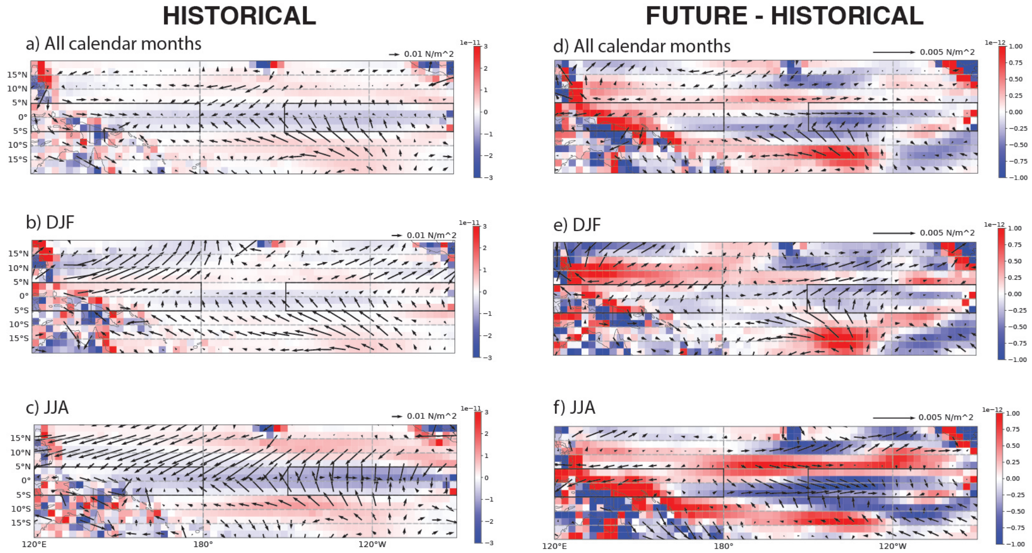

Figure S13: **Differences in wind stress (vectors) and wind stress divergence (colors) between high and low gradient sensitivity models, as defined in the main text.** a)-c): Differences over the historical period, for all calendar months (a), DJF (b), and JJA (c). d)-f): Future-historical differences, for all calendar months (d), DJF (e), and JJA (f).

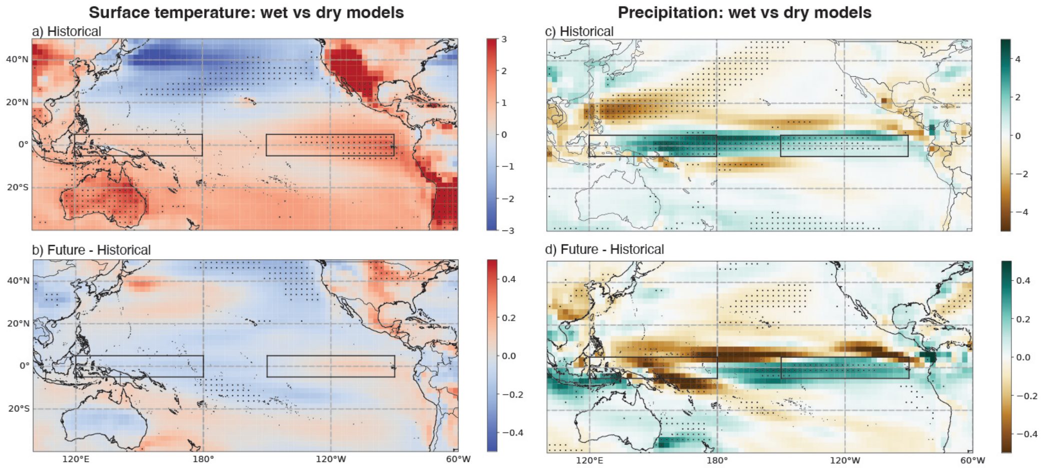

Figure S14: **Mean-state differences between wet and dry models, as defined in the main text (see also Table S6).** a) 20th century surface temperature, b) 21st century - 20th century surface temperature, c) 20th century precipitation, d) 21st century - 20th century precipitation. In all panels, differences are calculated as wet - dry model averages, for the relevant time period or epoch difference. Stippling indicates locations where model populations differ at the 90% level, as computed using a Mann-Whitney U test.

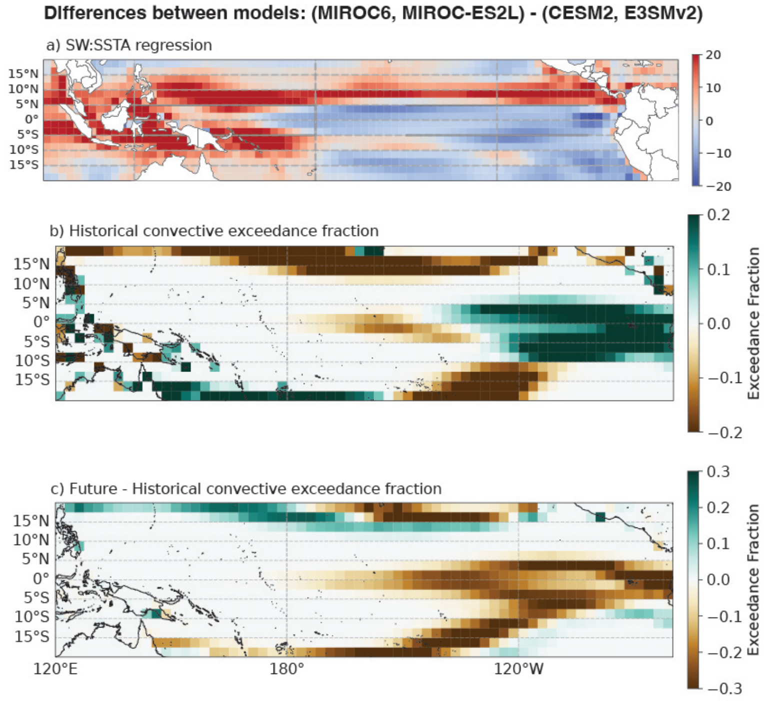

Figure S15: **Feedback differences between models**. Behavior of shortwave feedback and convective exceedance fraction, differenced between the wettest two models (MIROC6 and MIROC-ES2L) and the next-wettest (CESM2 and E3SMv2). a) Gridpont regression of net shortwave flux on SSTA, over the 20th century. b) Convective exceedance fraction over the 20th century. c) Convective exceedance fraction change, 21st century - 20th century.

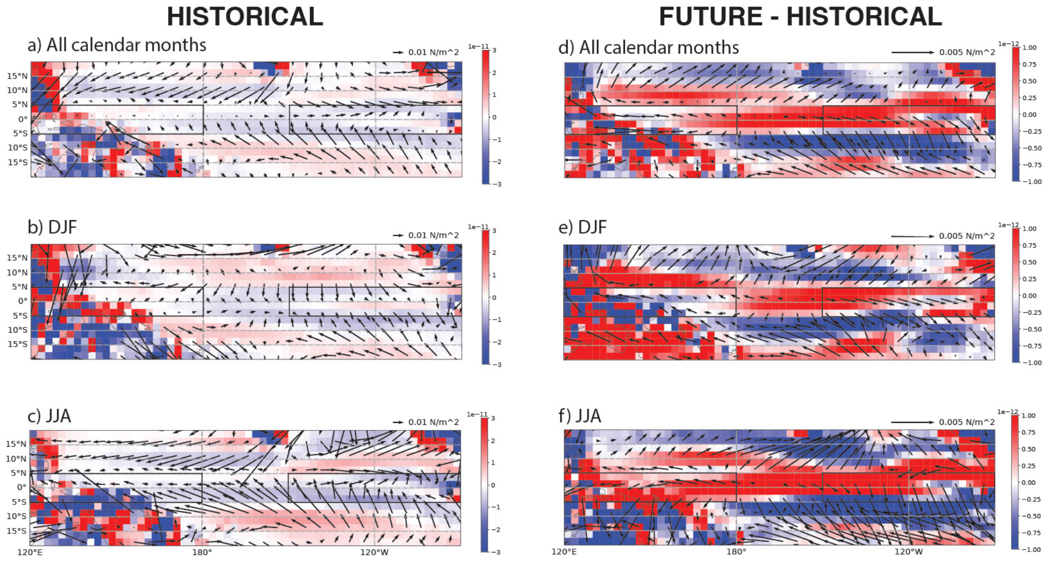

Figure S16: **Differences in wind stress (vectors) and wind stress divergence (colors) between sets of 'wet' models: (MIROC6, MIROC-ES2L) - (CESM2, E3SMv2).** a)-c): Differences over the historical period, for all calendar months (a), DJF (b), and JJA (c). d)-f): Future-historical differences, for all calendar months (d), DJF (e), and JJA (f).

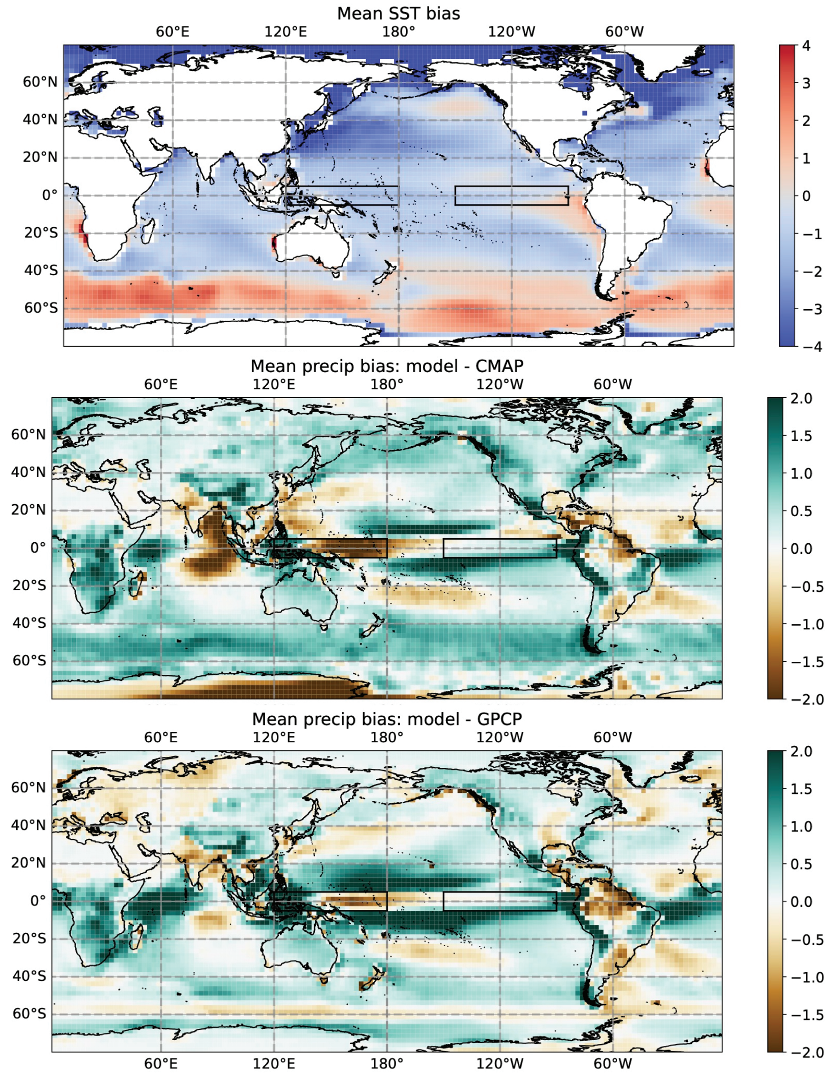

Figure S17: **Model bias.** Multi-model mean 20th century bias in a) SST (°C), b) precipitation (CMAP), and c) precipitation (GPCP). Units for precipitation are mm/day. Bias maps are computed over the 1979-2024 period; ERSSTv5 is used for SST (little difference was seen between bias calculated with different SST datasets).

Supplementary Tables

Table S1: **SMILE ensemble sizes in the present analysis.** Numbers indicate the ensemble size associated with each set of external forcing factors. [a]*EC-Earth: SSP5-8.5 data used only for members corresponding to a historical simulation*

Model name	Historical	SSP5-8.5	SSP3-7.0	SSP2-4.5	SSP1-2.6
ACCESS-ESM1-5	40	40	40	40	40
CanESM5	40	25	25	25	25
CESM2	100	15	100	16	
E3SMv1	20		20		
E3SMv2	20		20		
EC-Earth3[a]	25	15			
GFDL-SPEAR	30	30			
IPSL-CM6A-LR	33	7	11		
MIROC6	50	50	3	50	50
MIROC-ES2L	30	10	10		

Table S2: **SMILE variables employed in the present analysis.** X indicates the presence of the specified field in the available databases. SLP = sea level pressure; τ_u/τ_v = zonal/meridional wind stress; Φ_{500} = 500 hPa geopotential height.

Model name	Temp	Precip	SLP	τ_u	τ_v	Φ_{500}	Evap
ACCESS-ESM1-5	X	X	X			X	X
CanESM5	X	X	X	X		X	X
CESM2	X	X	X	X	X	X	X
E3SMv1	X	X	X	X	X	X	X
E3SMv2	X	X	X	X	X	X	X
EC-Earth3	X	X	X	X	X	X	X
GFDL-SPEAR	X	X	X			X	
IPSL-CM6A-LR	X	X	X	X	X	X	X
MIROC6	X	X	X	X	X	X	X
MIROC-ES2L	X	X	X	X	X	X	X

Table S3: Δ_Δ **for all models considered in the present analysis**. $\Delta_\Delta$computed as the epoch difference in ΔSST for (2050-2100) - (1950-2000) normalized to the global-mean temperature epoch difference over the same periods. Units are C/C. Data from all calendar months is used for all calculations. "Uncertainty" indicates the difference between the maximum and minimum Δ_Δ estimates, generated for each available emissions scenario. A value of N/A for this parameter indicates that only a single emissions scenario was available for that model.

Model name	Sensitivity	Uncertainty
ACCESS	0.166	0.047
CanESM5	0.094	0.008
CESM2	0.313	0.092
E3SMv1	0.091	N/A
E3SMv2	0.163	N/A
EC-Earth3	0.086	N/A
GFDL-SPEAR	0.134	N/A
IPSL-CM6A	6.09×10^{-4}	0.002
MIROC6	0.230	0.033
MIROC-ES2L	0.142	0.021

Table S4: **Seasonally varying** Δ_Δ **for all models considered in the present analysis**. Same as Table S3, but for Δ_Δ values averaged over DJF or JJA

Model name	Sens. (DJF)	Uncert. (DJF)	Sens. (JJA)	Uncert. (JJA)
ACCESS	0.127	0.052	0.191	0.034
CanESM5	0.058	0.014	0.147	0.016
CESM2	0.322	0.127	0.358	0.109
E3SMv1	0.108	N/A	0.098	N/A
E3SMv2	0.158	N/A	0.190	N/A
EC-Earth3	0.044	N/A	0.126	N/A
GFDL-SPEAR	0.148	N/A	0.093	N/A
IPSL-CM6A	0.074	0.008	-0.063	0.019
MIROC6	0.262	0.044	0.205	0.037
MIROC-ES2L	0.182	0.022	0.124	0.011

Table S5: **Linear ΔSST trend estimates over 1950-2100**. Trends are calculated using the ensemble mean, normalized to the global-mean temperature trend over 1950-2100. Units of trend are C/decade/C.

Model name	Trend	Uncertainty
ACCESS	0.0148	0.0034
CanESM5	0.00844	0.00056
CESM2	0.0292	0.0095
E3SMv1	0.00812	N/A
E3SMv2	0.0145	N/A
EC-Earth3	0.0081	N/A
GFDL-SPEAR	0.012	N/A
IPSL-CM6A	-7.32×10^{-5}	0.00031
MIROC6	0.020	0.0028
MIROC-ES2L	0.0126	0.0017

Table S6: **List of ensembles which are members of the different model groupings mentioned in the main text: 'high-sensitivity', 'low-sensitivity', 'wet', and 'dry'.** Group membership is indicated by an X in the relevant column for each model.

Model name	High-Sens.	Low-Sens.	Wet	Dry
ACCESS	X			X
CanESM5		X		X
CESM2	X		X	
E3SMv1		X		X
E3SMv2	X		X	
EC-Earth3		X		X
GFDL-SPEAR				X
IPSL-CM6A		X		X
MIROC6	X		X	
MIROC-ES2L			X	
